# Assigning sex to pre-adult stalk-eyed flies using genital disc morphology and *X *chromosome zygosity

**DOI:** 10.1186/1471-213X-6-29

**Published:** 2006-06-16

**Authors:** Martin Carr, Samuel Cotton, David W Rogers, Andrew Pomiankowski, Hazel Smith, Kevin Fowler

**Affiliations:** 1 Department of Biology, University of York, PO Box 373, York, YO10 5YW, UK; 2 Department of Biology, University College London, Wolfson House, 4 Stephenson Way, London, NW1 2HE, UK; 3 Wolfson Institute for Biomedical Research, University College London, The Cruciform Building, Gower Street, London, WC1E 6BT, UK

## Abstract

**Background:**

In stalk-eyed flies (Diopsidae) the eyes and antennae are laterally displaced at the ends of elongated eyestalks. Eyespan and the degree of sexual dimorphism in eyespan vary considerably between species and several sexually dimorphic species show sexual selection through female mate preference for males with exaggerated eyespan. The genes on which selection acts to regulate eyespan remain to be identified. This could be achieved by comparing gene expression during eyestalk development in males and females if the sex of pre-adult flies could be reliably assigned. Here we describe two techniques, one morphological and one microsatellite-based, that identify the sex of stalk-eyed fly larvae and pupae.

**Results:**

We showed that genital discs of the stalk-eyed fly *Teleopsis dalmanni *have two highly distinct morphologies, compact ("C") and lobed ("L"). Segment composition (revealed by Engrailed expression) was consistent with C morphology being typical of males and L morphology of females. We confirmed the proposed association between disc morphology and sex by evaluating the combined heterozygosity of four *X*-linked microsatellite markers. We demonstrated that individuals with C genital discs had hemizygous (male) genotypes while those with L discs were heterozygous (female) genotypes. Similar dimorphism in genital disc morphology was observed in eight other species spanning three representative Diopsid genera. In every case the segment composition supported C morphology being male and L morphology female. We assigned larval sex by C or L morphology and compared cell division frequencies in male and female eye-antennal discs in two species (*T. dalmanni *and *Diasemopsis meigenii*) sexually dimorphic for eyespan. The number of mitotic (anti-H3-labelled) cells did not differ between the sexes in either species.

**Conclusion:**

We have made novel use of two complementary techniques for identifying the sex of pre-adult stalk-eyed flies. These procedures will facilitate studies of the evolution of sexually dimorphic development in a variety of other species. Morphology and En expression in male and female genital discs are highly conserved within each genus of Diopsidae. Finally, sexual dimorphism for eyespan in two Diopsid species is unlikely to be due to an increased rate of cell division at the third larval instar in males.

## Background

Understanding the evolution of sexually dimorphic development is a key goal in many biological contexts [1-3]. However, in many taxa the ability to investigate dimorphism in early gene expression and development is impeded by the lack of a reliable method for assigning sex to embryos, larvae or other early life stages. For example, in holometabolous insects adult morphology is determined largely prior to formation of the primary sex organs and eclosion. Investigation of sexually dimorphic development would be greatly facilitated by the identification of sex in the absence of traditional cues such as gonads or genitalia.

Hypercephaly, in the form of lateral extensions of the head capsule, is observed in several families of Diptera [4]. A particularly exaggerated form is found in the Diopsidae (stalk-eyed flies) in which eyes and antennae are laterally displaced at the end of eyestalks in both sexes. In many Diopsid species eyespan (the distance between the eyes) is sexually dimorphic, with males being larger than females. Eyespan and the degree of sexual dimorphism in eyespan varies considerably within the family [5,6], and there is empirical evidence that the highly exaggerated eyestalks found in males have evolved under sexual selection through strong female mate preference for males with larger eyespan [7-10]. Eyespan is determined prior to eclosion and is sensitive to external stress during pupation (e.g. heat shock; [11]). Developmental studies indicate that the expression of key regulatory genes involved in early head capsule specification is similar to that observed in other Dipterans [12-14].

From an evolutionary standpoint there is considerable interest in identifying the timing of expression of the hitherto unknown genes on which selection acts to regulate eyespan in sexually dimorphic Diopsid species. One potentially powerful approach is to compare gene expression during the development of eyestalks in between the sexes, since sexual dimorphism likely results from differential gene expression with respect to sex. However, this requires a method for determining the sex of pre-adult flies. In the model Dipteran *Drosophila melanogaster *it is possible to do this either on the basis of gonad size (the male gonad being significantly larger than the female in 3^rd ^instar larvae), or by using *X*-linked genetic markers such as the cuticle pigmentation gene yellow. Neither method is applicable in stalk-eyed flies in which the larval gonads of both males and females are equally small and undifferentiated and visible genetic markers are lacking. Here we present an alternative, novel method based on genital disc morphology and *X*-linked DNA markers, which can be applied in principle to many different Diopsid species, and potentially to other taxa.

The adult structures of Dipteran flies, including stalk-eyed flies, develop from the larval imaginal discs, which originate as invaginations of the embryonic ectoderm. In *D. melanogaster *it has been shown that with the exception of the eye-antennal and genital discs all of the imaginal discs are composed of cells derived from a single embryonic segment [15]. Imaginal disc cells multiply throughout larval development and differentiate during metamorphosis. While the other imaginal discs exist as pairs, in *Drosophila *the genital disc is a single disc in each sex [16].

In *Drosophila *the genital disc develops into the internal and external genitalia and the analia, collectively known as the terminalia. It is the only imaginal disc with an unambiguous sexually dimorphic morphology. The genital discs of both sexes comprise cells from the eighth, ninth and tenth/eleventh abdominal embryonic segments and the fate of these cells depends on the fly's sex [17,18]. In male larvae, the cells of the eighth segment have repressed proliferation, as these cells would otherwise develop into the female genitalia; while in female larvae, cells from the ninth segment, which would otherwise give rise to the male genitalia, do not develop. In both sexes cells from the tenth/eleventh abdominal segments develop into the analia [19].

In the model stalk-eyed fly species, *Teleopsis dalmanni *(previously known as *Cyrtodiopsis dalmanni*; [20]), we found that the genital discs have two distinct morphologies. We have termed these "compact" and "lobed" morphologies to reflect the fact that the former morph is relatively flat and compact. In *D. melanogaster*, segment polarity genes such as *engrailed *(*en*) have very different expression patterns in the male and female discs, which reflect later differences in eventual segment fate [21]. The pattern of *en *protein expression in *T. dalmanni *genital discs showing the compact morphology resembled that observed in the male genital discs of *D. melanogaster*. As in *D. melanogaster*, male Diopsids have a single *X *chromosome whilst females have a pair [22]. We confirmed the proposed association between disc morphology and sex by evaluating the combined heterozygosity of four *X*-linked microsatellite markers; individuals with a compact form of genital disc had hemizygous (male) genotypes, whereas individuals with lobed discs tended to exhibit heterozygous (female) genotypes.

To determine the extent to which male and female genital disc structure and segmental composition is conserved across the Diopsid clade, we characterised morphology and EN expression in a panel of Diopsid species spanning three genera. A compact/lobed dimorphism similar to that of *T. dalmanni *was observed and in all species studied EN expression patterns were consistent with the compact morphology being typical of males across genera.

We exploited our ability to assign sex by genital disc morphology to compare cell division rates in male and female eye-antennal discs. In *T. dalmanni *the larger eyespan of males is due, at least in part, to the eyestalks being composed of more cells. This could reflect a higher rate of cell division or a prolonged period of proliferation in males. We used genital disc morphology to determine whether the frequency of cell division differs between male and female H3 antibody-labelled eye-antennal discs in two sexually dimorphic species, *T. dalmanni *and *Diasemopsis meigenni*. We found no significant differences in dividing cell counts between the sexes in either species.

## Results

### Genital disc morphology in *D. melanogaster *and *T. dalmanni*

Most of the imaginal discs in *D. melanogaster *are essentially two-dimensional with folds in the plane of the disc epithelium. In contrast, the genital disc epithelium is folded into a fully three-dimensional structure with distinct ventral and dorsal sides [21]. By the third larval instar the morphology of the male and female genital imaginal discs is clearly dimorphic. Both discs are bilaterally symmetrical. The male discs have a somewhat flattened structure. The posterior half of the disc contains the repressed female primordia on the ventral side and the anal primordia on the dorsal side. Both primordia have undergone relatively little proliferation and form single layers of cells with little folding. The anterior of the disc consists of the male primordia, which have grown to form a series of concentric folds. The female discs have a complex folded structure reflecting the outgrowth of the left and right female primordia on the ventral side of the disc. Both the repressed male primordia and the anal primordia lie on the dorsal side of the disc and, having undergone relatively little proliferative growth, form a single thickened layer.

**Figure 1:**
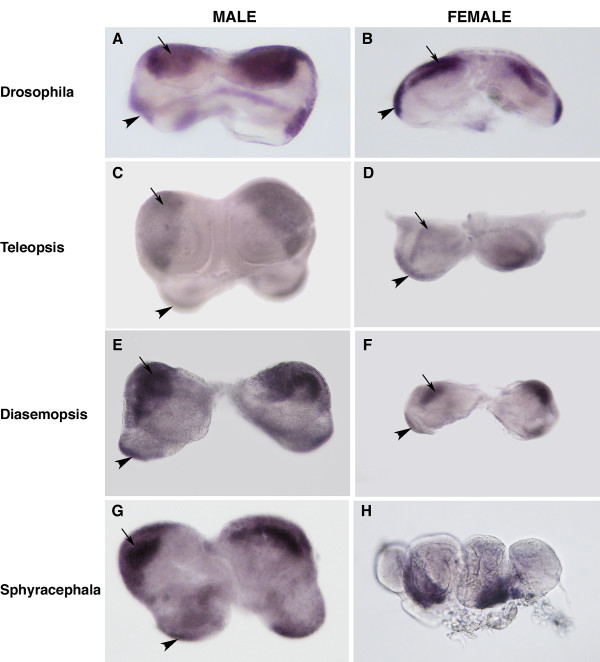
Genital disc morphology and Engrailed (EN) expression in *D. melanogaster *and representative species of three Diopsid genera: *Teleopsis*, *Diasemopsis *and *Sphyracephala*. All discs are bilaterally symmetrical. **A. **EN expression in male third instar genital disc (ventral view) of *D. melanogaster*. Note EN expression in two posterior lateral domains (arrowhead indicates the posterior domain on the left side of the disc) derived from the posterior compartments of the tenth abdominal segment. The larger anterior domains (arrow indicates the anterior domain on the left side of the disc) are derived from the posterior compartment of the ninth segment and give rise to parts of the male genitalia in the adult. **B. **EN expression in female third instar genital disc (ventral view) of *D. melanogaster*. As in the male disc, EN is expressed in two posterior lateral domains (arrowhead indicates the posterior domain on the left side of the disc) derived from the posterior compartments of the tenth abdominal segment. The larger anterior domains (arrow indicates the anterior domain on the left side of the disc) are derived from the posterior compartment of the eighth segment and give rise to parts of the female genitalia in the adult. **C, E & G. **Male third instar genital discs of *T. dalmanni *(**C**), D. *meigenii *(**E**) and *S. beccarii *(**G**). **D, F & H. **Female third instar genital discs of *T. dalmanni *(**D**), D. *meigenii *(**F**) and *S. europaea *(**H**). With the exception of the female discs in *Sphyracephala*, morphology and EN expression appear similar to that of the male and female discs in *Drosophila*. On each side of the disc EN is expressed in two domains, one anterior (arrows) and one more posterior and lateral (arrowheads). Anterior is uppermost in all panels.

In both male and female discs the three primordia (male, female and anal) are contiguous. As each is derived from a different segment, their boundaries can be revealed by staining the discs with an antibody raised against a highly conserved epitope of the *en *protein product, the expression of which is restricted to the posterior compartments of all segments. In the male disc, EN expression in the male primordium forms a broad anterior band on both ventral and dorsal sides of the discs. On the dorsal side two lateral stripes representing the anal primordium are visible, while a small central patch corresponding to the posterior compartment of the repressed female primordium is visible ventrally (Figure 1A). In the female disc, EN is expressed in one ventral anterior band at the boundary of the female and repressed male primordium, a dorsal anterior band at the boundary of the repressed male and anal primordium and in two laterally placed stripes along the dorsal posterior edges of the disc revealing the location of the posterior compartments of the left andright anal primordium (Figure 1B).

In the stalk-eyed fly, *T. dalmanni*, two distinct genital disc morphologies can be observed. One type, which we have termed "compact", resembles the morphology of the male genital disc in *Drosophila*. The posterior half of the disc has a relatively simple flattened structure while the anterior forms a series of concentric folds. EN is expressed in two posterior strips similar to the staining of the anal primordium in *Drosophila *and in two large anterior patches, which could correspond to male primordium expression (Figure 1C). The other type, which we have termed "lobed", has a complex folded structure with the left and right discs connected only by a thin bridge of tissue. Bands of EN expression are visible dorsally and laterally (Figure 1D).

### Genital disc morphology and microsatellite genotypes

We used a microsatellite-based approach to verify the sex of the compact (putatively male) and lobed (putatively female) genital discs of *T. dalmanni*. A panel of six *X*-linked microsatellite markers were assayed for the 1989 and 1993 populations and four markers showing the most variation were selected for further analysis. In order to determine the accuracy of assays using these loci to distinguish reliably between the sexes in *T. dalmanni*, a trial was conducted using adult males and females. Using combined data from the 4 loci, 91.7% of males (n = 12) were hemizygous at all four loci and 90% of females (n = 20) were found to carry two different alleles for at least one marker locus (see Table 2).

**Table 1:** Microsatellite loci used in this study; repeat motif, allele size range in base pairs, and nucleotide sequence (all from [24]).

*Locus*	*Repeat Motif*	*Product Size (bp)*	*Primer Sequence*
ms-054	[AC]2ATTAT[AC]10AT[AC]1	165	F:FAM-ACGGAAGTAACACAAAAAGAT R:TCAGCGCTACTCACAGAACTAACT
ms-125	[GT]14	153	F:FAM-TGGTGTTAATGAACGAGTGACTTC R:TGCCATTCATGCAAGTCTTC
ms-167	[AC]1TC[AC]9	222	F:FAM-GCTGCGAGCTGTAAAACAGA R:GGCAGTGACAATGGCAGTAA
ms-395	[GT]10AT[GT]1	200	F:HEX-CGAGTAGAGCACTTTGAAGATACA R:TTGCGGTTGTAGAAGTTTGC

**Table 2:** Test of association between number of alleles at each of four *X*-linked microsatellite loci and sexual identity for adults of known sex in *T. dalmanni*. Loci which could not be successfully genotyped are denoted by a dash. Twelve males and twenty females were assayed.

*Sex*	*ms-054*	*ms-125*	*ms-167*	*ms-395*	*Sex*	*ms-054*	*ms-125*	*ms-167*	*ms-395*
M	1	2	2	1	F	2	2	2	1
M	1	-	-	-	F	2	1	2	2
M	1	1	1	1	F	2	1	2	1
M	1	1	1	1	F	-	1	2	1
M	-	1	1	1	F	1	1	2	1
M	1	1	1	1	F	-	1	1	1
M	1	1	1	1	F	2	1	2	1
M	1	1	1	1	F	2	2	2	1
M	1	1	-	1	F	2	1	2	1
M	1	1	-	1	F	2	2	2	1
M	1	1	1	1	F	2	2	2	1
M	1	1	1	1	F	2	2	2	1
					F	2	1	2	1
					F	2	2	2	1
					F	1	2	2	1
					F	2	2	2	1
					F	2	2	2	1
					F	1	1	1	-
					F	1	1	2	2
					F	1	1	2	-

A further assay based on the same 4 loci was used to test for an association between the sex of a larva (inferred on the basis of their number of alleles per microsatellite locus) and the morphological type of genital disc. Genital discs were dissected from 3^rd ^instar larvae, stained with methylene blue and typed for morphology (compact or lobed). DNA for microsatellite analysis was extracted from the remaining larval tissue of 16 with the compact type of genital disc and 16 individuals with the lobed type (Table 3). Individuals with the compact disc type were found always to have only one allele at each genotyped microsatellite. With the exception of one individual who could be assayed at only 3 of the loci, the members of the lobed disc type invariably had two alleles for at least one marker locus. A contingency test revealed a highly significant association between type of disc morphology and *X *chromosome zygosity level (χ^2 ^= 28.69, df = 1, *P *< 0.001). The expected values for this test have been adjusted using the observed error levels from the genotyping trials of adults. Note that even if this error level is omitted from the contingency test, the significant association between disc morphology and level of *X *chromosome zygosity remains (χ^2 ^= 28.25, df = 1, *P *< 0.001). Thus the microsatellite genotyping confirms the inferences from the comparative morphology and *en *expression analyses that individuals with compact genital disc morphology are male and those with lobed morphology are female.

**Table 3:** Test of association between sex of larvae (inferred on the basis of their number of alleles per microsatellite locus) and morphological type of genital disc in *T. dalmanni*. Loci which could not be successfully genotyped are denoted by a dash. Sixteen larvae with "compact" (C) genital disc morphology and 16 with "lobed" (L) genital disc morphology were assayed.

*Disc type*	*ms-054*	*ms-125*	*ms-167*	*ms-395*	*Sex*	*Disc type*	*ms-054*	*ms-125*	*ms-167*	*ms-395*	*Sex*
C	-	-	-	1	M	L	2	1	1	1	F
C	1	-	1	1	M	L	-	1	2	1	F
C	1	1	1	1	M	L	2	1	1	1	F
C	1	1	1	1	M	L	1	1	1	2	F
C	1	1	1	1	M	L	2	1	-	1	F
C	1	1	1	1	M	L	2	2	1	1	F
C	1	1	1	1	M	L	2	1	2	1	F
C	-	1	1	1	M	L	2	1	2	1	F
C	1	1	-	1	M	L	2	2	1	1	F
C	1	1	1	1	M	L	2	1	1	2	F
C	1	1	1	1	M	L	2	2	2	1	F
C	1	1	-	1	M	L	1	2	2	2	F
C	1	1	1	1	M	L	2	2	2	1	F
C	1	1	1	1	M	L	2	2	2	1	F
C	1	1	1	1	M	L	2	2	2	1	F
C	-	1	1	1	M	L	1	1	-	1	M

### Genital disc morphology among Diopsid species

**Figure 2:**
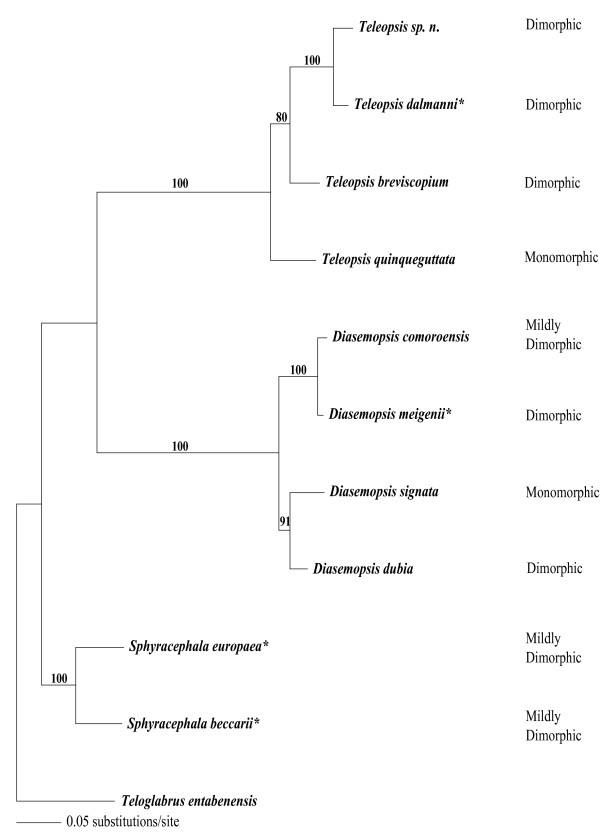
Distance-based neighbour-joining tree of the Diopsid species clade, constructed using sequences of *white *and *wingless*. The tree has been rooted with the sequence from the non-hypercephalic species, *T. entabenensis*. Representative species of each genera that were analysed in this study and featured in Figure 1 C-H are asterisked. Bootstrap values are positioned on the nodes and are percentages taken from 1,000 replicates. The degree of sexual dimorphism in eyespan is indicated for each species.

We investigated the morphologies and *en *protein expression patterns of the male and female discs in a further eight species sampled from three representative genera of Diopsids in order to characterise morphological variation among the clade. Three further *Teleopsis *species were chosen (*T. breviscopium, T. quinqueguttata *and *T. species n*) to supplement our findings described above for *T. dalmanni*. Also we selected three more distantly related *Diasemopsis *species (*D. meigenii*, *D. comoroensis *and *D. dubia*). The *Sphyracephalae *have been shown to form a basal genus within extant Diopsidae [23] and so finally we analysed two species from this genus (*S. beccarii *and *S. europaea*). Examples of representative species from each genus are presented in Figure 1C–H and their position within the clade of Diopsid species is shown in Figure 2.

In all species, two distinct morphologies of genital disc with associated EN patterns were found (examples shown in Figure 1C–H). In the species sampled we found that discs from one genus could be distinguished from those of the other two on the basis of subtle morphological differences. Within each genus, morphology and en staining patterns of both types of disc were effectively identical in all species. In *Teleopsis *species, we can assign sex according to genital disc morphology with some confidence given the consistent results of our microsatellite-based assay to identify the male and female disc types in one genus member, *T. dalmanni *(Figure 1C and 1D).

In the *Diasemopsis *species, one of the disc morphologies (putatively male) showed an almost identical pattern of EN expression to that seen in male *Teleopsis *discs (Figure 1E). One slight difference was that the right and left discs were connected by only a thin band of tissue in *Diasemopsis *while nearly fused in *Teleopsis*. The other disc morphology and EN patterns observed were very similar to those of the female discs in the *Teleopsis *species (Figure 1F). We, therefore, putatively identify this morphology as female.

In the basal Diopsids, one disc type closely resembled the morphological and *en *protein expression patterns of male discs found in *Teleopsis *species (Figure 1G). The other disc had a unique morphology consisting of four loosely connected lobes (Figure 1H). Given the strong conservation of male patterns, the former disc morphology is assumed, putatively, to be male and, by exclusion, the latter unusual morphology is assumed to be female in this species.

### Cell division rates of males and females during prepupal growth

Counts of mitotic cells in the eye-antennal discs at the prepupal stage were obtained for males and females of two sexually dimorphic Diopsid species (*T. dalmanni*, n = 8 males and 5 females; *D. meigenii*, n = 6 males and 9 females). Cell counts were non-normally distributed and therefore analysed by non-parametric methods (Wilcoxon test). In both species, cell counts did not differ significantly between the sexes (*T. dalmanni*, χ^2 ^= 3.09, df = 1, *P *= 0.08; *D. meigenii*, χ^2 ^= 0.06, df = 1, *P *= 0.81).

## Discussion

Analysis of the development of sexually dimorphic characters requires a method for identifying the sex of pre-adult individuals. Here we describe two different techniques one morphological and one microsatellite-based that can be used to identify the sex of stalk-eyed fly larvae and pupae. However, these procedures will likely be of use to investigators studying the evolution of sexually dimorphic development in a variety of other species. We also highlight some of the uses and applications of these techniques.

### Complementary approaches to determining sex of pre-adult flies

The morphological approach relies on the strong sexual dimorphism we observe in genital disc morphology (two highly distinct forms, compact and lobed), present in all Diopsid species examined. However, it is not intrinsically obvious whether these forms reflect sexual identity. A comparative argument can be made based on the similarities between stalk-eyed fly disc morphologies and those of the male and female discs in *Drosophila *but, given the evolutionary distance between Diopsids and Drosophilids, independent verification of the sex of each disc type is desirable. *X*-linked microsatellite genotyping [24], correctly identified the sex of adult stalk-eyed flies in over 90% of cases. The level of accuracy could be further increased by the use of additional microsatellite loci or by using flies of greater heterozygosity. A single adult male was heterozygous at two of the *X*-linked microsatellite loci. This was unexpected and potential explanations include contamination of the genomic DNA or a duplication of the region of the *X *chromosome containing ms-125 and ms-167, with each duplicated locus evolving independently.

Each method, morphological or genetic, has advantages and disadvantages. Assays of genital discs in order to assign sex are only possible for a limited period during development. For example, in *D. melanogaster*, sexual dimorphism in genital disc morphology is not apparent until the third larval instar and it becomes progressively more difficult to observe the genital disc as metamorphosis proceeds. In contrast, the use of microsatellites has the advantage over simple observation of genital disc morphology as they can be used to identify the sex of individuals at any growth stage. However, there are constraints to the adoption of a microsatellite-based approach, as it is also more costly, more time-consuming and currently less reliable than morphological observation; in Diopsids, *X*-linked microsatellites are currently limited to only a few species within the *Teleopsis *genus [24].

In the three genera we have studied at least one of the discs has proved almost identical in terms of morphology and EN expression to either the male or the female disc in *T. dalmanni*. We are therefore confident that the morphological assay can be used to identify the sex of larvae from these species. In the absence of *X*-linked microsatellite markers it may be possible to verify this by *in vivo *culture of male and female discs, as has been achieved for analysis of eye-antennal discs in *T. dalmanni *[13].

### Genital disc variation within the Diopsidae

We have described differences between the genital discs of males and females in three stalk-eyed fly genera. EN expression patterns and morphology are very highly conserved within Diopsid genera and, with one notable exception, quite similar to that described in *D. melanogaster*. EN expression patterns are consistent with the hypothesis that the genital discs are derived from more than one embryonic segment and suggest that as in *D. melanogaster*, the posterior part of each disc gives rise to the analia and the anterior to the genitalia. It should be possible to verify these assumptions by *in vivo *culture of male and female discs.

Male discs are highly conserved within and between Diopsid genera. The female discs of *Teleopsis *and *Diasemopsis *are similar in appearance, suggesting a conserved morphology since the two lineages diverged. Female discs in the phylogenetically basal *Sphyracephala *exhibit a more complex morphology quite unlike their counterparts in the other Diopsid genera or in *D. melanogaster *so that it is unclear which regions of the disc represent the genital and anal primordia. Although we have not tried to assign functionality to these differences, our data provide a firm basis for future studies of the evolution of genital disc morphology and sexual dimorphism in stalk-eyed flies.

### Evolution of genital disc morphology in Schizophoran flies

Phylogenetically the Schizophoran dipterans are traditionally viewed as comprising two sister groups, the acalypterates and calypterates [25,26]. Genital discs have been characterised in a pair of calypterate species and in each case individuals exhibit three genital discs. One disc comprises cells from the ninth, tenth and eleventh abdominal embryonic segments and a further pair of laterally situated discs are derived from cells displaced from the eighth abdominal segment (*Calliphora erytrocephala *[27]; *Musca domestica *[28,29]). In contrast, the acalypterate *D. melanogaster *possesses a single genital disc [16]. Our study of *T. dalmanni *shows that for a second acalypterate species, individuals of each sex possess one genital disc and lateral genital discs are absent. Two recent studies have established that the acalypterate Diopsidae are the basal family within Schizophorans based on morphological [30] and molecular phylogenies [31]. Consequently it can be inferred that a single genital disc is the likely ancestral form of Schizophoran flies and possession of a pair of lateral discs is a derived feature of calypterate flies. However, further studies of genital disc morphology in other Schizophoran species such as the Centrioncidae [32,33], or outgroup families from the lower Cylcorrapha [34] are needed to confirm this inference.

### Cell division rates of males and females during prepupal growth

The cellular basis of the sexual dimorphism for eyespan in *T. dalmanni *may be due, at least in part, to differences in cell number. We have exploited our ability to identify larval sex in this species to compare rates of cell division in male and female eye-antennal discs. The difference between the number of mitotic H3-labelled cells in males and females at the prepupal stage was not significant, suggesting that the rate of cell division is similar in both sexes at this timepoint. We are currently developing techniques for assessing cell division at subsequent stages of metamorphosis, when the developing eyestalks are less accessible to experimental manipulation.

## Conclusion

We describe the novel application of two complementary techniques, one morphological and one microsatellite based, for identifying the sex of pre-adult stalk-eyed flies. Both procedures will aid studies of the evolution of sexually dimorphic development in Diopsid and other flies and the microsatellite-based approach may be applied to a wide range of species. To demonstrate the potential value of our methods, we used them to assign larval sex in a study comparing cell division frequencies in male and female eye-antennal discs of two sexually dimorphic species. Our findings indicate that sexual dimorphism for eyespan in Diopsids is unlikely to be due to an increased rate of cell division in males at the end of the third larval instar.

## Methods

### Fly stocks and rearing methods

Experimental samples of *T. dalmanni *for this study were obtained from a pair of laboratory populations originally derived from field collections in 1989 and 1993 near the Gombak River in Malaysia and maintained subsequently as independent stocks. To minimise the potential effects of inbreeding, both stocks have been maintained in population cages containing at least 200 individuals, kept at at 25°C on a 12 hour: 12 hour light: dark cycle, and fed ground sweetcorn. In order to maximise *X*-chromosomal heterozygosity in females when genotyping microsatellites, we analysed the hybrid offspring of males from the 1989 stock crossed to females of the 1993 stock.

A further six stalk-eyed fly species were used during this study. They were *T. quinqueguttata *(originally collected from Ulu Gombak, Malaysia), *T. species n *(Chiang Mai, Thailand), *T. breviscopium *(Bukit Timah, Singapore), *Sphyracephala beccarii *(Pietermaritzberg, South Africa), *S. europaea *(Szeged, Hungary), *Diasemopsis dubia *(Pietermaritzberg, South Africa), *Diasemopsis meigenii *(Pietermaritzberg, South Africa) and *D. comoroensis *(Mohéli, Comoros). All species were maintained under similar conditions to those of *T. dalmanni*.

The sample of wild-type *D. melanogaster *came from a stock established in 1972 from a collection in Dahomey (now Benin, West Africa). Subsequently this has been maintained on standard sugar-yeast food medium at large population size in cage culture at 25°C and exhibits high levels of genetic variation [35].

### Imaginal disc preparation

Third instar *T. dalmanni *larvae were dissected in 1 × PBS, with the larval cuticle being inverted to expose the genital disc. Discs were stained in 0.1% methylene blue (Fisher Scientific) for 5 minutes and fixed in 1% gluteraldehyde (Sigma) in 1 × PBS, before being removed from the cuticle wall and scored for their morphology.

### Immunohistochemistry

Antibody staining was performed using the protocol of [12]. For *D. melanogaster *and the suite of stalk-eyed fly species, third instar genital discs were labelled with a 1:20 dilution of mouse monoclonal anti-EN mAb 4F11 antiserum (gift from N. Patel). *T. dalmanni *eye-antennal discs from the early pupal stage were labelled with a 1:1000 dilution of rabbit polyclonal anti-phospho-Histone H3 (Ser10) antiserum (Upstate). Labelled discs were mounted in 90% glycerol and photographed using a Nikon Coolpix 4500 digital camera attached to a Leica DMLB microscope. Labelled cells from each eye disc (excluding the portion of connecting tissue between the eye and antennal discs) were counted from the photographs.

### DNA extraction

Tissue from individual adults or larvae was ground in TNES (50 mM Tris pH 8.0, 400 mM NaCl, 20 mM EDTA, 0.5% SDS), to which Proteinase K (1 mg/ml) was added. The mixture was incubated overnight at 37°C. 5 M NaCl was then added and the DNA was precipitated with absolute EtOH.

### PCR

*X *chromosome zygosity levels were determined by genotyping individuals for the *X*-linked microsatellite loci ms-054, ms-125, ms-167 and ms-395 (described in Wright et al. 2004 and see Table 1) Amplification was performed in 10 μl volume reactions (1 μl template DNA, 0.5 U DNA polymerase (Abgene), 2.5 mM MgCl_2_, 1.3 mM forward primer labelled with a fluorescent dinucleotide and 19.7 mM reverse primer). PCR was initiated with a two minute denaturing step at 94°C, followed by thirty cycles of denaturing at 94°C for 30 seconds, annealing at 60°C for 30 seconds and extension at 72°C for 45 seconds. The final step was a ten minute extension at 72°C. Microsatellite genotyping was carried out using a 3100 DNA Analyzer (Applied Biosystems) and analysed with Genescan 3.1.2 software (Applied Biosystems).

## Authors' contributions

MC conceived the study, contributed to the design and execution of the experiment, and helped to draft the manuscript. SC participated in the execution of the experiment and helped to draft the manuscript. DR contributed to the design of the experiment. KF conducted the statistical analysis. HS, AP and KF jointly conceived the study with MC, contributed to design and coordination, and drafted the manuscript. All authors have read and approved the final manuscript.
